# The Effect of Accentuation of Curve of Spee on Masticatory Efficiency—A Systematic Review and Meta-Analysis

**DOI:** 10.3390/children10030511

**Published:** 2023-03-04

**Authors:** Zainab A. Alkhalaf, Mohammed Ghazi Sghaireen, Rakhi Issrani, Kiran Kumar Ganji, Norah Nasser Alruwaili, Raghad Mohammed Alsaleh, Musab Redha S. Alruwaili, Meshari Farhan Alabdali, Munahi Abdullah Rushdallah Alsirhani, Mohammad Khursheed Alam

**Affiliations:** 1 Department of Prosthetic Dentistry, College of Dentistry, Jouf University, Sakaka 72345, Saudi Arabia; 2 Department of Preventive Dentistry, College of Dentistry, Jouf University, Sakaka 72345, Saudi Arabia; 3 Department of Periodontics & Implantology, Sharad Pawar Dental College, Datta Meghe Institute of Higher Education & Research, Sawangi (Meghe), Wardha 442107, India; 4 Dental Intern, College of Dentistry, Jouf University Sakaka, Sakaka 72341, Saudi Arabia; 5 Department of Dental Research Cell, Saveetha Institute of Medical and Technical Sciences, Saveetha Dental College and Hospitals, Chennai 600077, India; 6 Department of Public Health, Faculty of Allied Health Sciences, Daffodil lnternational University, Dhaka 1216, Bangladesh

**Keywords:** Curve of Spee, dentofacial anatomy, masticatory efficiency, occlusal curvature, Curve of Wilson

## Abstract

Background: The appropriate alignment of the lower teeth is indicated by the Curve of Spee (COS), which can be observed in the sagittal profile view of human skulls. Graf Von Spee made the initial observation on this occlusal curvature. Through this systematic review and meta-analysis, we evaluated studies that looked at how COS affected masticatory activities. Methods: The databases PubMed-MEDLINE, Web of Science, Cochrane, and Scopus were all searched. A total of 12 documents were ultimately picked because they met the necessary inclusion and exclusion requirements. The data was then loaded into the RevMan 5 programme for meta-analysis after being chosen for information on the sample size, variables analyzed, and various aspects of the research. Results: The Curve of Spee was found to have a noticeable impact on both the masticatory efficiency as well as dentofacial alignment in the 12 studies that we selected for the review and meta-analysis. In addition, other occlusal curvatures such as the Curve of Monson and the Curve of Wilson were found to be of vital importance on a similar level to the COS. The meta-analysis further revealed that seven of the included clinical trials had mentioned the noticeable impact on masticatory efficiency. Conclusions: This study focused on the significance of the COS on force distribution in the oral cavity as well as the necessity of COS corrections after receiving full orthodontic care. Following orthodontic treatment, the COS, along with other occlusal curves such as the Curve of Wilson and the Curve of Monson, is essential in removing strains from the condyle, as well as the maxilla and mandible, which enhances masticatory effectiveness and lessens the overall strain on a patient’s oral cavity.

## 1. Introduction

Orthodontic remedial treatment is increasingly expensive but essential in many people’s lives. To rectify malocclusions in the dentition, the primary goal is to align the teeth and jaws [1]. Given how commonplace it is in the contemporary Western world, one might wonder why we do not observe the need for this correction in the vast majority of the ancient world. Although there may have been the occasional case of a misaligned orofacial complex in the past, it is now fairly rare for a post-industrial adolescent to not need any kind of dental intervention. The link between this and nutrition is strongly supported by the data. Increased bite force and chewing cycles brought on by a harsh or abrasive diet during growth leads to a flatter Curve of Spee (COS), more sub-nasal prognathism, and more dental attrition [1,2]. Because these characteristics encourage better dental and skeletal occlusion, the current post-industrial diet causes an epidemiological transition in occlusion, which substantially increases malocclusion in modern populations.

The Curve of Spee (COS), which is visible in the sagittal profile image of human skulls, indicates the exact alignment of the lower teeth. Graf Von Spee made the initial remark in 1890 [2]. Recent research has revealed that it had a curvature that extends from the centre teeth’s incisor edges to their distobuccal cusps [3]. The literature claims that the mesial inclination of the lower molar teeth’s long axis caused the masticatory muscles to function improperly, which led to the development of the COS [4,5,6]. One study suggested that the Curve of Spee may play a biomechanical role in the processing of food by enhancing the crush/shear ratio between the posterior teeth and the efficiency of occlusal pressures during the masticatory process [4]. This suggestion alluded to the idea of “masticatory efficiency.” The COS permits unrestricted lower jaw motions while enhancing food chewing by maintaining upper and lower tooth contacts throughout operation [2]. By pursuing an aesthetic grin, the COS also enhances face aesthetics [7]. There are two main ways to evaluate the COS. The anterior Condyle border, the buccal cusps of the second and first molars, the cusp tips of the canine and premolars, and the incisor edge of the two central incisors are all included in the first illustration of the inferior radius of a circle [8]. The second method of COS assessment was built on the occlusal plane as well as the width and depth of the posterior teeth’s arches [9]. Yet there is disagreement on how to quantify the COS. Some authors did not quantify COS using the lower incisors [10]. Thanks to recent technological developments and the use of a three-dimensional (3D) scanner, COS measurement when using a digital cast analysis is now easier and more accurate. Previously, all measurements were performed using a divider, calipers, and a coordinate apparatus on hard study models or pictures of the models [11]. To accomplish the proper incisor relation and posterior teeth occlusion during full orthodontic treatment, the COS of the mandibular teeth must be levelled [12,13]. However, there is still debate over the COS levelling methods used to increase the stability of orthodontic outcomes [14]. We must also consider the disadvantages pertaining to an accentuation in the COS. An accentuated Curve of Spee can create occlusal interferences between the upper and lower teeth, which can cause discomfort, pain, and damage to the teeth. This can further lead to malocclusion, which means that the upper and lower teeth do not fit together properly and can cause problems with mastication, phonation, and oral hygiene, and can increase the risk of tooth decay and gum disease. An accentuated curve of Spee can also put excessive stress on the TMJ, which is the joint that connects the jaw to the skull. This can lead to TMJ disorders, which can cause pain, clicking, popping, and limited range of motion in the jaw. This also increases the risk of bruxism, which is a condition where people grind or clench their teeth unconsciously. This can lead to tooth wear, jaw pain, headaches, and other complications. Moreover, an accentuated Curve of Spee can make orthodontic treatment more challenging, as it can require more complex movements to correct the malocclusion, issues which can be resolve by carefully flattening of the COS as recommended by certain studies [14,15,16]. Regardless of the effect on the mandible, the Temporomandibular Joint (TMJ), or even the lower teeth, since 1972 Andrews [15] has recommended levelling the COS to a flat surface in order to assist in the formation of an ideal occlusion. Kanavakis and Mehta [16] discovered a connection between the flatness of the COS and the TMJ sound.

In order to determine whether the COS had any observable effects on the occlusal curvature/malocclusion in the oral cavity as well as the effects of the COS on enhancing masticatory functions, the aim of this systematic review and meta-analysis was to examine the available literature. The primary variable that we examined through the studies that we chose for review and later meta-analysis was the impact of the COS on masticatory efficiency.

## 2. Materials and Methods

This review protocol was registered in the International Prospective Register of Systematic Reviews (PROSPERO; registration number: CRD42023389852).

### 2.1. Protocols Employed

The Orderly Reviews in Health Care: Meta-Examination book and the Preferred Reporting Items for Systematic Review and Meta-analysis (PRISMA) criteria were followed in the conduct of this systematic review [17].

### 2.2. Review Hypotheses

By the means of this systematic review and meta-analysis, we assessed studies that ascertained whether the COS had any discernible impacts on the occlusal curvature/malocclusion in the oral cavity as well as the effects of the COS on enhancing masticatory functions. The effect of the COS on masticatory efficiency was the main variable that we examined through the papers that we chose for review and subsequent meta-analysis, and this was the main goal of the study. 

### 2.3. Inclusion Criteria

Articles that featured material relevant to the review’s objectives and covered all age groups were chosen for full-text screening. We considered including articles that presented randomized/non-randomized investigations, systematic reviews with large sample sizes, in-depth case reports, and validated questionnaire-based studies.

### 2.4. Exclusion Criteria

Studies involving animal subjects, seminar presentations, academic publications, opinion pieces, and incomplete data were not included in the scope of our systematic examination.

Our search did not limit the selection of studies based on their year of publication; instead, we considered all publications that had been released in connection with our topic (where the number of papers itself was found to be quite sparse in number). This is a result of the dearth of literature on the topic that is pertinent to the objective of our inquiry.

Studies using placebos were not included in the study. All reviews of the literature and cases that were written in languages other than English were also disregarded.

### 2.5. Data Selection and Coding

Two independent reviewers searched through pertinent papers in various databases and online search engines using MeSH terms such as “Curve of Spee,” “Dentofacial anatomy,” “Masticatory efficiency,” “Occlusal curvature,” and “Wilson’s curve.” The chosen papers were compared, and a third reviewer was consulted if there was a dispute.

The same two reviewers separately extracted the following information after selecting the articles: author, year of publication, nation, type of publication, study topic, population demographics (n, age), outcome measure(s), pertinent result(s), and conclusion(s). A third reviewer was consulted once the data were compared to go through any discrepancies.

### 2.6. Study Selection

**Figure 1:**
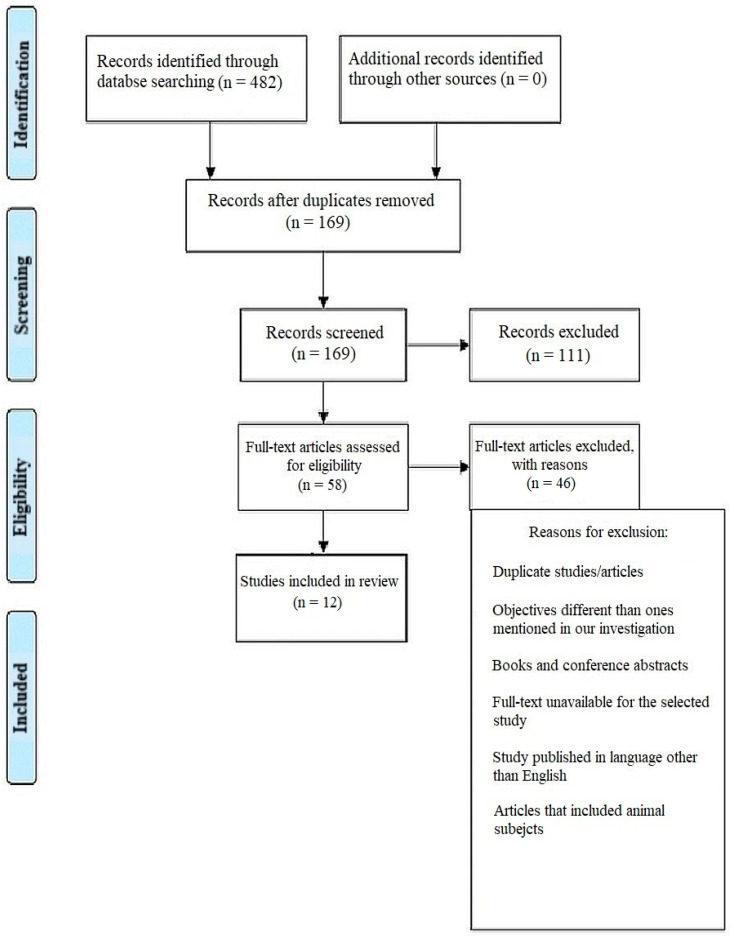
Representation of selection of articles through PRISMA framework.

A thorough search of the online journals turned up 482 documents in total, and 169 of the papers were first selected. After removing 111 articles that were identical to or duplicates of each other, only 58 original papers remained. In total, 46 further articles were excluded after the abstracts and titles of the submissions were examined. Ultimately, 12 documents—mostly in-vitro experiments, literature reviews, and comparative analyses—were chosen that satisfied the essential inclusion and exclusion criteria. (Figure 1) Using relevant keywords, reference searches, and citation searches, the databases PubMed-MEDLINE, Web of Science, Cochrane, and Scopus were all searched. “Curve of Spee,” “Dentofacial anatomy,” “Masticatory efficiency,” “Occlusal curvature” and “Wilson’s curve” were the search terms used to search the database. The keywords were used as filters in the above-listed databases. 

### 2.7. Statistical Analysis

**Figure 2:**
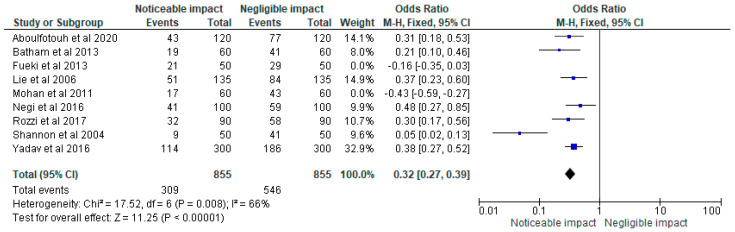
Forest plot of the odds ratio representing the impact status of COS accentuation on masticatory efficiency in the clinical trials [2,18,19,20,21,22,23,24,25] selected for our meta-analysis (assuming a 95% confidence interval).

**Figure 3:**
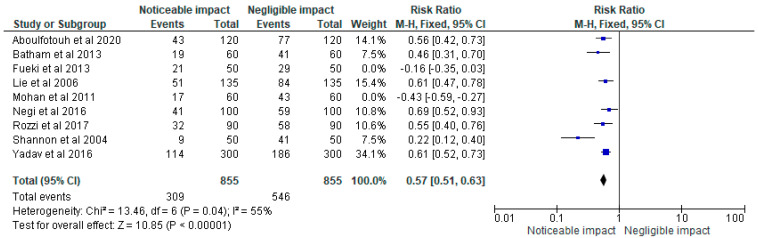
Forest plot of the risk ratio representing the impact status of COS accentuation on masticatory efficiency in the clinical trials [2,18,19,20,21,22,23,24,25] selected for meta-analysis (assuming a 95% confidence interval).

**Figure 4:**
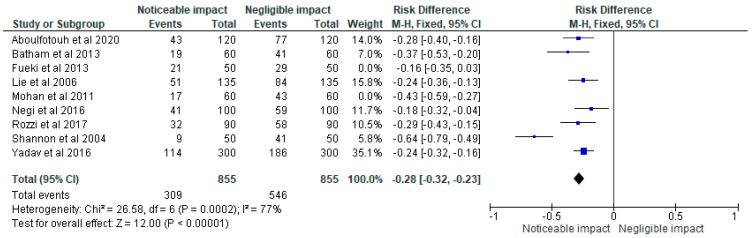
Risk difference representing the impact status of COS accentuation on masticatory efficiency in the clinical trials [2,18,19,20,21,22,23,24,25] selected for meta-analysis on a forest plot (assuming a 95% confidence interval.

The data was then loaded into the RevMan 5 programme for meta-analysis after being chosen for information on the sample size, variables analyzed, and various aspects of this research. As part of the meta-analysis for our investigation, forest plots showing the odds ratio, risk ratio, and risk difference (using a fixed effects model) of the different treatment modalities were generated. These plots are given in Figure 2, Figure 3 and Figure 4 respectively assuming a 95% confidence interval.

### 2.8. Risk of Bias Assessment

The AMSTAR-2 approach was used to evaluate the studies we chose for Table 1 for bias [26]. In addition to a number of other instruments that have served the same goal, AMSTAR 2 has been made accessible as a critical evaluation tool for systematic reviews. As seen in Table 2 below, it is a 16-point checklist. Two extremely noticeable devices served as the foundation for the creation of the first AMSTAR tool. The original AMSTAR was duplicated by two new instruments. The AMSTAR 2 risks of bias items identify the domains specified in the Cochrane risk of bias instruments for systematic reviews. These demonstrate that a decision was made in each instance after feedback from more than 30 methodological specialists.

## 3. Results

The various characteristics of the studies selected after implementation of the requisite inclusion/exclusion criterion, such as sample size, mean age of the study participants, study objectives, and their respective inferences/outcomes [2,18,19,20,21,22,23,24,25,27,28] are displayed in Table 2.

The odds ratio, risk ratio, and risk difference, respectively, as determined by the meta-analysis of the various treatment methods indicated in the studies chosen for our systematic review, are shown in Figure 2, Figure 3 and Figure 4.

## 4. Discussion

The relationship between an individual’s occlusal curvature and the masticatory strain brought on by nutrition during development is obvious. Changes in prehistoric populations, contemporary people, and studies using animal models all reflect this. Mandibular and dental traits might reveal information about the types of diet ingested during a person’s growth and development [29]. Understanding the causes of the aesthetic and health problems that result from malocclusion today depends critically on understanding secular patterns in malocclusion. We can improve our understanding of how the COS controls not only masticatory efficiency but also other aspects of occlusion by taking an anthropological approach to dentofacial misalignment in the archaeological record and in the present.

The effect of the COS on masticatory efficiency was the primary variable on the basis of which the meta-analysis was conducted. All the studies concerning the review objectives were considered except for studies by Fueki et al. [20] and Mohan et al. [23], since the study done by Fueki et al. [20] was primarily concerned with investigating the link between occlusal curvature and the ability of young adults with permanent teeth to mix and comminute food using a masticatory performance test that did not directly assess a relationship between the COS and mastication. As for the study by Mohan et al. [23], the participants were primarily divided into two distinct age groups without mentioning/taking into account the pre/post differences of COS correction on occlusal movement. Additionally, the lack of a proper follow-up record made it ineligible for it to be included in the meta-analysis. As such, these two studies were reviewed but they carried no weight in the meta-analysis as evident in the forest plots.

The number of participants in each of the studies has been included in the meta-analysis, with the effect of the COS on masticatory performance assessed by the number of noticeable and negligible events in each of the trials. The risk of bias pertaining to the mta-analysis is moderately high, which can be attributed to the heterogeneity percentage, which happens to be fairly high for a systematic review such as this, but the studies that we reviewed as per our objectives were varied in their methodologies and, although the variable of our concern was examined in all of them, the study designs and protocols that they followed were somewhat different from one another.

The majority of occlusal macrowear, it is commonly believed, is produced during chewing food, with the exception of people who grind their teeth out of habit [30]. In a 1999 study, Sengupta and associates [31] examined the COS, third molar occurrence, and occlusal macrowear. When compared to normal pre-industrialized dentitions, the modern dentition is linked to a higher frequency of third molar impaction and agenesis, as well as mild wear rates [30,32,33]. Because of this, the authors postulate that historically high rates of wear brought on by abrasive diets kept the COS flat. Their argument that any damage in the current era is caused by sustained use rather than by abrasive diets is probably a sound one [30]. The findings, however, showed that there was no direct correlation between the COS and macrowear; the COS was not sustained by wear. Although the authors sought to ascertain if a deeply arched COS should be incorporated into dental prosthetic designs, this evidence can potentially be interpreted in other ways. Given that the mandibular structure is often established by the time the dentition exhibits considerable wear, it is unlikely that this wear will have a significant impact on COS depth. Given this, it is plausible to infer that mastication of food is the common source of both wear and the COS. The mandibular morphology is altered to produce a flatter COS by chewing abrasive and difficult foods during growth; if a similar diet is followed in adulthood, more severe wear may be visible. COS depth and tooth wear are probably connected, although perhaps not in the way that Sengupta and colleagues have suggested [31].

Dietary consistency changes have an impact on occlusion because they influence orofacial morphology. This is due to its effect on chewing, which ultimately has an impact on the biomechanical stress placed on the jaw. The strain is affected by the differential between difficult and abrasive foods. Tough foods, such as meat, require more bite force and more chewing cycles before swallowing, whereas abrasive foods, such as nuts, produce more friction and wear with each bite [34,35]. Because it measures how hard the muscles must work to chew food, bite force is significant [35]. The growth and preservation of alveolar bone are impacted by the tension caused by bite force. The fact that a deeper curve might enhance the force potential for each chewing cycle provides proof that the COS has a role in determining biting force potential [20]. As determined by a single occlusion of the opposing dental arcades, a chewing cycle is the movement of the food bolus around the mouth [20]. Certain foods take longer to digest, which has an impact on how much muscle is used and how the orofacial bones are stimulated. There is proof that a person’s biological capacity for performing each of these characteristics is significantly influenced by their jaw morphology. Greater sub-nasal prognathism has been linked to the capacity to carry out more chewing cycles at a greater biting force. This is made possible by a stronger jaw and a more acute gonial angle [20,34]. This combination of characteristics would improve occlusion and result in higher levels of tooth macrowear. Diet has a big impact on these bone and muscle elements, which are crucial for growing and maintaining a normal dental complex.

In a geometric-morphometric investigation, Laird and colleagues [1] correlated the COS and prognathism in contemporary humans and archaic Homo Sapiens. As a whole, contemporary humans were more orthognathic, although there are still variations amongst populations, according to their findings. People with a deeply curved COS are orthognathic, but those with a flattened COS were more prognathic. The COS and alveolar prognathism co-varied [1]. Although their analysis is thorough, they may want to reconsider their conclusion that the COS and prognathism are population-specific markers in modern humans [1]. Metric measurements and a qualitative review of bite alignment may be some of these characteristics. There is reason to suppose that by using traits such as these in conjunction with other dietary reconstruction techniques, one can deduce details about the diet ingested during development, though this would need further testing to be confirmed [36]. This is useful in supporting assertions about nutrition and food processing made on the basis of stable isotope studies and archaeological interpretations because it is not always obvious how a group subsisted.

The fact that we did not assess the other occlusal curvatures (such as the Curve of Monson and the Curve of Wilson) as well as the COS could be deemed to be one of the flaws of our systematic review, since the various curvatures combine to influence the dentofacial anatomy and other aspects of malocclusion. Furthermore, our meta-analysis did not include a lot of studies with a higher sample size than what could be considered ideal and that might pose a question upon the credibility of the results and might have introduced a certain degree of bias as far as the meta-analysis is concerned. Hence, we recommend more studies that assess the various occlusal curvatures individually so that their impact on the patient undergoing any form of orthodontic treatment is clearly evaluated and clinically implemented.

## 5. Conclusions

The importance of the COS on force distribution in the oral cavity and the requirement for COS corrections following comprehensive orthodontic treatment were the main subjects of this investigation. Following orthodontic treatment, the COS is crucial in removing strains from the condyle, as well as the maxilla and mandible, which improves masticatory effectiveness and reduces the overall strain on a patient’s oral cavity. This is true of other occlusal curves as well, such as the Curve of Wilson and the Curve of Monson, although they were not assessed as rigorously as the COS for the sake of the major objectives of our investigation.

## Figures and Tables

**Table 1:** AMSTAR-2 16-point checklist of risk of bias assessment in studies selected for the review.

*Studies Selected*	*Question and Inclusion*	*Protocol*	*Study Design*	*Comprehensive Search*	*Study Selection*	*Data Extraction*	*Excluded Studies Justification*	*Included Study Details*	*Risk of Bias*	*Funding Sources*	*Statistical Methods*	*Risk of Bias in Meta-Analysis*	*Risk of Bias in Individual Studies*	*Explanation of Heterogeneity*	*Publication Bias*	*Conflict of Interest*
Aboulfotouh et al., 2020 [18]	Yes	Yes	Yes	Yes	Yes	No	No	No	Yes	N/A	Yes	Yes	Yes	Yes	Yes	Yes
Batham et al., 2013 [19]	Yes	Yes	Yes	Yes	Yes	No	No	No	Yes	N/A	Yes	Yes	Yes	Yes	Yes	Yes
Fueki et al., 2013 [20]	Yes	Yes	Yes	Yes	Yes	No	No	No	Yes	N/A	Yes	N/A	Yes	Yes	Yes	Yes
Hasan et al., 2021 [27]	Yes	Yes	Yes	Yes	Yes	No	No	No	Yes	N/A	Yes	Yes	Yes	Yes	Yes	Yes
Lie et al., 2006 [21]	Yes	Yes	Yes	Yes	Yes	No	No	No	Yes	Yes	Yes	Yes	Yes	Yes	Yes	Yes
Marshall et al., 2019 [28]	Yes	Yes	Yes	Yes	Yes	No	No	No	Yes	Yes	Yes	Yes	Yes	Yes	Yes	Yes
Mohan et al., 2011 [22]	Yes	Yes	Yes	Yes	Yes	No	No	No	Yes	N/A	Yes	Yes	Yes	Yes	Yes	Yes
Negi et al., 2016 [23]	Yes	Yes	Yes	Yes	Yes	No	No	No	Yes	N/A	Yes	Yes	Yes	Yes	Yes	Yes
Yadav et al., 2016 [24]	Yes	Yes	Yes	Yes	Yes	No	No	No	Yes	N/A	Yes	N/A	Yes	Yes	Yes	Yes
Rozzi et al., 2017 [25]	Yes	Yes	Yes	Yes	Yes	No	No	No	Yes	N/A	Yes	N/A	Yes	Yes	Yes	Yes
Shannon et al., 2004 [2]	Yes	Yes	Yes	Yes	Yes	No	No	No	Yes	N/A	Yes	Yes	Yes	Yes	Yes	Yes

**Table 2:** Description and outcomes as observed in the studies selected for the systematic review.

Author and Year of Study	Study Design	Sample Size	Study Description/Characteristics	Study Outcome/Inference
Aboulfotouh et al., 2020 [18]	Retrospective study	120 patients (47 males)	This retrospective cross-sectional study sought to ascertain the Spee curve’s depth in various malocclusion forms. According to the type of malocclusion as described by the British criteria of classification, their casts were classified into 4 equal groups of 30 casts each.	The deepest Spee curves were seen in Class II Division 2 malocclusions, which were then followed by Class II Division 1, Class I, and Class III, which had the shallowest Spee curves. There was a sizable difference between Class I and both divisions of Class II malocclusion. The depths of the Spee curve in Class II Division 1 and Division 2 instances, as well as both divisions of Class II and Class III malocclusion, differed significantly. In the depths of the Spee curve, there was no obvious distinction between Class I and Class III malocclusion.
Batham et al., 2013 [19]	Observational study	60 patients (23 males)	The objective of this study was to assess the association between dentoskeletal morphology and the Curve of Spee in various skeletal growth patterns. Sixty orthodontic patients (17–26 years old) were gathered, and their pretreatment lateral cephalographs and dental casts were separated into three groups based on various skeletal growth patterns. Average, horizontal, and vertical growth patterns are represented by Groups A, B, and C, respectively. Measurements and statistical analysis of cephalometric and study cast factors were conducted using the Curve of Spee as the dependent factor and other variables as the independent variables. Four cephalometric measures, four linear and four angular, were taken. We measured four study cast properties.	Sagittal maxilomandibular differences affected the Curve of Spee in all three groups. ANB angle and SNB angle both positively and negatively linked with changes in the depth of the Spee curve. Dental characteristics including overjet, overbite, and Molar relation were strongly connected with the Curve of Spee. In individuals who were growing horizontally, a highly significant relationship was observed between rising Spee curve depth and an increase in overbite.
Fueki et al., 2013 [20]	Observational study	50 patients	The aim of this study was to investigate the link between occlusal curvature and the ability of young adults with permanent teeth to mix and comminute food. Using peanuts as the test food, a masticatory performance test was used to measure the ability to chew food, and the results were rated according to the median particle size.	A linear regression analysis that took into account the maximum bite force revealed that the occlusal curvature’s sphere radius was a significant predictor of both the median particle size and the mixing ability index. These results showed that food mixing and chopping skills were superior in people with a flatter curvature (larger sphere) in their mandibular arch. In young adults with permanent dentitions, occlusal curves such as the Spee curve seemed to be related to one’s ability to mix and cut food.
Hasan et al., 2021 [27]	In-vitro study	2 replica models	The effect of Curve of Spee (COS) flattening on stresses and displacement on several mandibular landmarks and lower teeth during posterior dental loading was examined in this work using three-dimensional (3D) finite element analysis. A three-dimensional hemi mandibular model was created using a genuine mandible of choice. The second premolar’s cusp tip was 2.4 mm deep and bent when the lower teeth were initially positioned. To verify the findings, a second duplicate with flat-aligned teeth was made by uprighting the premolars and molars. The mesiobuccal cusp of the lower first molar was loaded on both models, and the stresses and displacements that resulted on the lower teeth and mandibular landmarks were examined.	The Mesiobuccal cusp tip of the first molar displayed the highest stress values in models that were both flat and bent. Mesiodistally, the teeth displacement of the curve model was more than that of the flat model. The first and second premolars held the most distal displacement in the curve model. Because of the COS’s flattening, the stresses acting on the entire jaw were enhanced, and the lower teeth’s mesiodistal displacement was lessened.
Lie et al., 2006 [21]	Retrospective study	135 patients (50 males)	The goals of this study were to evaluate post-treatment Spee curve (CS) changes and predict post-treatment stability using cephalometric traits. On lateral cephalograms and study models of 135 subjects, the curve depth (CD), location of the deepest point (LDP), and eight cephalometric parameters were measured prior to receiving orthodontic treatment (T1 years), at the end of orthodontic treatment (T2 years), and at least 3 years after retention (T3 years). The sample was divided into lower arches that had been treated and those that had not. The upper arch was treated for all patients. Malocclusions that were 25% Class I, 73% Class II, and 2% Class III treated were included in the sample.	The results showed that unexpected changes were frequently occurring and that the post-treatment CD was frequently unstable. Between T1 and T2, the LDP migrated distally, and between T2 and T3, it showed a mesial displacement. The post-treatment changes in CD and LDP were evaluated using stepwise regression analysis, and it was found that a deep curve at T2 was associated with a reduction in CD from T2 to T3. The distal location of the LDP, proclination of the lower incisors at T2, and extraction therapy were all associated with mesial displacement of the LDP, which occurred between T2 and T3. The results also suggest that an optimal CD of about 2.0 mm at T2 was associated with the least amount of post-treatment change.
Marshall et al., 2019 [28]	In-vitro study	N/A	The purpose of this study was to test the hypothesis that tooth eruption, occlusal load, and mandibular movement interact to produce the Spee curve of the mandible and the compensating curve of the maxilla. A straightforward finite element model was used for this (FE model). The researchers used a straightforward finite element model made up of maxillary and mandibular “blocks” to simulate tooth eruption and forces that prevent eruption. Following that, the doctors tested the hypothesis that curved occlusal planes are formed as a result of the interaction between mandibular movement, occlusal load, and tooth eruption.	The results demonstrated that the contacting maxillary and mandibular block surfaces changed from flat to curved when rhythmic chewing motion simulation, tooth eruption inhibition, and application of these simulations combined were used. The depth of the curvature appeared to be influenced by the radius of the mandibular block’s rotating (chewing) motion. The results also suggested that the function of the mandible and the interaction between the maxilla and mandible may have contributed to the evolution of the occlusal curvature in humans.
Mohan et al., 2011 [22]	Comparative study	60 patients	This study compared the disclusion in the premolar and molar area during protrusion to the Spee curve in human permanent healthy dentitions in two age groups. Sixty subjects were picked, equally split into two age ranges: 18–24 and 35–44. To determine the Spee curve, a digital camera was used to take pictures of the left side of the mandibular dental castings. An arc was formed by marking and connecting the canine cusp, the first molar’s mesiobuccal cusp, and the second molar’s distal cusp. The radius for this arc was calculated using the AUTOCAD software. To gauge the degree of posterior disclusion during edge-to-edge protrusion, a hard bite registration material was used to create a protrusive interocclusal record.	With ageing, the radius of the Spee curve did not change considerably. Age-related changes in the mean disclusion values in the premolar and molar regions were not statistically significant. Thus, it was concluded that the Spee curve flattened with age and that the disclusion values during protrusion decreased along with it.
Negi et al., 2016 [23]	Observational study	100 patients	This study sought to determine whether there was any sexual dimorphism by examining how the Curve of Spee, overjet, and overbite varied across Class-I and Class-II malocclusion participants. In total, 100 regular patients’ dental casts and the pre-treatment orthodontic patients’ records made up the study’s study material. The depth of the curvature of the Spee was used to separate the subjects into three groups. Consideration was also given to overjet and overbite.	Overall results of the current study revealed that deep Spee groups had much more overjet and overbite than normal groups, which affected their ability to masticate. These findings were supported by the study’s correlation coefficient. The subject’s gender had no discernible impact on the variables examined.
Yadav et al., 2016 [24]	Observational study	300 patients	This study’s primary goal was to examine the skeletal and dental correlations in people with different Spee curve depths in the vertical and horizontal planes. In total, 300 patients had their dental casts and lateral cephalograms obtained before treatment. The individuals were split into three groups based on the Spee depth curve. Group A (N = 100) represents the flat Spee curve, or less than 2 millimetres; Group B (N = 100) represents the normal Spee curve, or between 2–4 mm; and Group C (N = 100) represents the deep Spee curve, or more than 5 millimetres. On the lateral cephalogram, four angular and four linear measurements were made. On dental casts, the depth of the Spee curve, the overbite, the overjet, and the molar relation were measured.	The three groups’ differences in overjet in horizontal relationships were discovered to be statistically significant. It was discovered that the difference in molar relationships between Groups A, B, and C was significantly significant. There were no statistically significant differences in SNA, SNB, ANB, or beta angle between the three groups. The above three groups’ overbite differences were discovered to be statistically significant. It was discovered that none of the vertical skeletal parameters were statistically significant. In patients with a deep Curve of Spee, there was an increase in overjet, overbite, and Class II Molar Relation (Group C). Changes in the depth of the Spee curve had no effect on either the horizontal or vertical skeletal characteristics.
Rozzi et al., 2017 [25]	Retrospective study	90 patients (39 males)	This study’s objective was to ascertain how participants treated with preadjusted appliances in various skeletal vertical patterns responded to the levelling of the Spee curve. In total, 90 white individuals with a Spee curve of at least 2 mm before therapy made up the study sample. Their vertical face types were used to divide them into three categories. The various dental movements after treatment were assessed using cephalometric measures. Digital dental impressions were used to measure the Spee curve.	There were no observable variations in the skeletal traits between the three groups. For the dentoalveolar variables, the low-angle group showed significant buccal movements and mandibular incisor intrusion. The high-angle group showed more extrusion of the back teeth as well as an uprighting of the first and second molars. As a result, it was discovered that levelling of the Spee curve occurred in high-angle individuals by extrusion and up-righting of the posterior teeth and in low-angle patients through buccal movement and intrusion of the mandibular incisors.
Shannon et al., 2004 [2]	Retrospective study	50 patients (24 males)	The aim of this study was to evaluate changes in the Spee curve during treatment and their effects on dentofacial structures, identify factors influencing the stability of the Spee curve following treatment, and identify skeletal and dental patterns relevant to the depth of the Spee curve prior to treatment. Prior to, following, and at least two years after retention, patients’ lateral cephalogram and dental casts were inspected. The second teeth and premolars in the mandible had all emerged and were fully occluded in all individuals.	Between male and female patients, or between the right and left sides, there were no appreciable differences in pretreatment curve depth. In comparison to the non-extraction cases, the amount of curve relapse was not significantly different. Curve relapse rates were also correlated with increased overbite, irregularity score, and patients wearing detachable retainers. After therapy, there was only a 16% relapse of the levelling curve, and the Spee curve was largely stable.

## Data Availability

The data set used in the current study will be made available on request from Zainab A. Alkhalaf; dr.zainab.alkhalaf@jodent.org.
